# Genomic Recombination and Genetic Diversity of Emerging Novel Duck Orthoreovirus in China, 2018–2023

**DOI:** 10.1155/tbed/7746288

**Published:** 2026-11-05

**Authors:** Chenchen Jiang, Rongchang Liu, Bing Li, Wenzhe Liu, Di Lei, Yu Huang, Chunhe Wan, Yi Tang, Rendong Fang, Hongzhi Wang

**Affiliations:** ^1^ College of Veterinary Medicine Southwest University Beibei Chongqing China swu.edu.cn; ^2^ Fujian Provincial Key Laboratory for Avian Diseases Control and Prevention Institute of Animal Husbandry and Veterinary Medicine of Fujian Academy of Agricultural Sciences Fuzhou Fujian China; ^3^ Institute of Animal Sciences of Chinese Academy of Agricultural Sciences Haidian Beijing China caas.cn

**Keywords:** genetic evolution, novel duck orthoreovirus, pathogenicity, recombination

## Abstract

Novel duck orthoreovirus (NDRV) represents a major pathogen severely affecting the waterfowl farming industry in China, yet its evolutionary dynamics and reassortment patterns remain poorly defined. In this study, we sequenced the full genomes of 10 NDRV isolates obtained from Shandong and Jiangsu provinces (2018–2023) and analyzed their genetic features through sequence identity, phylogenetics, and reassortment detection. We also evaluated pathogenicity in 1‐day‐old ducklings for three representative isolates via intravenous injection. Our results show that nucleotide (nt) homologies across all gene segments ranged from 86.9% to 99.9%, and the L2, M1, S2, and S4 segments displayed greater sequence variability than the traditionally hypervariable S1 gene in our isolates. We identified recombination events in the M2 segment of strain XT18 (with SDYC and HN5d as parental strains) and the S4 segment of strain LY20 (with GX‐Y7 and SDYC as parents), which, to our knowledge, represent the first reports of recombination in the M2 and S4 segments of NDRV. In pathogenicity tests, strain SDYC induced the most severe splenic lesions and maintained the highest viral loads in vivo. Collectively, these findings clarify the molecular evolution and reassortment landscape of currently circulating NDRV strains and offer a scientific basis for selecting vaccine candidates and designing precision control strategies.

## 1. Introduction

A novel duck orthoreovirus (NDRV) has emerged as a major threat to China’s waterfowl industry, and its ongoing spread and evolution undermine the sector’s sustainability [1]. This virus primarily affects ducklings, causing splenic enlargement and necrosis, hepatic hemorrhagic necrosis, and tarsal joint swelling, with relatively high morbidity and mortality [2–4]. More critically, NDRV can trigger immunosuppression, which increases the risk of secondary infections and leads to growth retardation, vaccination failure, and reduced feed conversion efficiency; even recovered ducks often show persistent growth retardation syndrome [5]. A key turning point came in 2017, when variant strains such as XT18 began driving significantly higher morbidity and mortality [2].

Taxonomically, NDRV belongs to the family Reoviridae, genus *Orthoreovirus*. The virions are icosahedral, nonenveloped, and have a double‐layered capsid [6]. Its genome consists of 10 segments of double‐stranded RNA (dsRNA), grouped by molecular weight into large (L1–L3), medium (M1–M3), and small (S1–S4) [7, 8]. Each segment carries highly conserved terminal sequences (5^′^‐GCUUUU and 3^′^‐UCAUC‐5^′^) that are essential for transcription and replication [9]. Like avian orthoreovirus (ARV), the S1 gene is the most variable and functionally complex, with a unique tricistronic structure encoding three proteins, σC, p10, and p17. Of these, σC serves as the major outer capsid protein and type‐specific antigen, responsible for receptor binding and able to induce neutralizing antibodies, making it a key target for genetic analysis and recombinant vaccine development [10, 11]. As for antigenic properties, NDRV shares group‐specific antigens with ARV, but the antigenic relationships among different isolates remain unclear.

The genetic evolution of NDRV is driven primarily by two molecular mechanisms, genetic drift via point mutations and genetic shift via gene segment reassortment [12]. These act together to drive continuous viral change. Although currently circulating strains maintain relatively high nucleotide (nt) identity (typically ≥ 85%) [7, 13], even minor variations in key genes can significantly affect biological properties. For example, mutations in core proteins involved in attachment, entry, or replication can alter virulence, and the 18‐amino acid (aa) deletion in the σC protein of HN5d correlates strongly with its high pathogenicity [14].

Reassortment is a typical feature of segmented RNA viruses [15], and the segmented genome of orthoreoviruses makes reassortment likely to occur during coinfection. Comparative genomic studies have documented reassortment across the *Reoviridae* in different hosts [16, 17]. For instance, the μB‐ and σC‐encoding genes of the classical duck orthoreovirus (DRV) isolate TH11 may have originated from reassortment between ARV and Muscovy DRV (MDRV) [18], while the German isolate D2533/4/1‐10 appears to have arisen from reassortment involving at least three different host‐derived viruses, including classical and novel waterfowl orthoreoviruses plus an unidentified ARV [19]. In NDRV, the L1 segment of isolate FJ19 was confirmed as a recombinant of strains 03G and J18 [20]. Notably, significant genetic linkage may exist among the gene segments encoding μB, σB, and σC proteins, with specific gene combinations exhibiting codistribution patterns with strain pathologies [21]. Despite the accumulation of whole‐genome sequencing data, the precise mechanisms by which particular gene combinations determine viral virulence and pathotype remain incompletely understood.

As NDRV continues to evolve, its host range expands, and the risk of cross‐species transmission poses new challenges [4, 13, 22]. Recent studies have isolated NDRV from geese, while goose orthoreovirus (GRV) and novel GRV (NGRV) can also infect ducks and cause disease, indicating that waterfowl orthoreoviruses have broad cross‐infection capacity [4]. More concerning is the emerging threat to chickens, as the FJ19 strain caused hepatic and splenic hemorrhage and necrosis in chickens, unlike earlier reports suggesting low pathogenicity in this host [20]. These observations suggest that NDRV is no longer restricted to its traditional host range, and that natural reassortment between waterfowl‐ and chicken‐origin orthoreoviruses may further promote gene exchange at the wild bird–domestic poultry interface [23], indicating that the underlying evolutionary drivers warrant further investigation.

In this study, we performed whole‐genome sequencing on NDRV field isolates and conducted phylogenetic analysis to clarify their evolutionary positions across different gene segments and identify potential genetic reassortment events. These data deepen our understanding of NDRV molecular epidemiology and provide a critical foundation for precise control strategies.

## 2. Materials and Methods

### 2.1. Clinical Samples and Virus Isolates

Between 2018 and 2023, spleen and tendon samples were collected from ducks with suspected disease aged 4–18 days on large‐scale farms in Shandong, Jiangsu, and other provinces for case diagnosis and pathogen isolation. Tissue samples that tested positive for NDRV by PCR were subjected to virus isolation in LMH (Leghorn male‐chicken hepatocellular‐carcinoma, ATCC CRL‐2117) cells following routine procedures, and 10 NDRV isolates were finally obtained. Reference strains of waterfowl‐origin orthoreoviruses were collected from the literature and public databases. Detailed information on the reference strains is provided in Table 1.

**Table 1:** Information of waterfowl‐derived orthoreovirus strains used in this study.

Category	Strain	Location	Time	Host	Accession n
DRV	091	China	2009	Pekin duck	JX478J250–JX478259
	TH11	China, Jiangsu	2011	Pekin duck	KC493571–KC493574 JX440512–JX440514 JX826588, JX826589, JQ664689
	HN5d	China	2013	Pekin duck	KT861587–KT861596
NDRV	XT18 ^∗^	China, Shandong	2018	Pekin duck	MK749398–MK749407
	SDYC ^∗^	China, Shandong	2018	Pekin duck	ON009427–ON009436
	SDLS ^∗^	China, Shandong	2018	Pekin duck	ON009437–ON009446
	GX‐Y7	China, Guangxi	2018	Pekin duck	MN747004–MN747013
	SY	China	2018	Cherry Valley duck	MK955818–MK955827
	SD19‐6201	China, Shandong	2019	Cherry Valley duck	MT829205–MT829214
	LRS‐GD20	China, Guangdong	2020	Muscovy duck	OM930745–OM930754
	ZSS‐FJ20	China, Fujian	2020	Muscovy ducks	OM930755–OM930764
	YL	China	2020	Duck	MZ733722–MZ733731
	SDWF21 ^∗^	China, Shandong	2021	Pekin duck	PZ829670–PZ829679
	LY20 ^∗^	China	2021	Pekin duck	ON040907–ON040916
	JSSQ22 ^∗^	China, Jiangsu	2022	Pekin duck	PZ829680–PZ829689
	JS0122 ^∗^	China, Jiangsu	2022	Pekin duck	PZ829690–PZ829699
	QAU‐001	China	2022	*Anas platyrhynchos*	OQ446451–OQ446460
	SDLH23 ^∗^	China, Shandong	2023	Pekin duck	PZ829700–PZ829709
	SDLC23 ^∗^	China, Shandong	2023	Pekin duck	PZ829710–PZ829719
	SDZB23 ^∗^	China, Shandong	2023	Pekin duck	PZ829720–PZ829729
MDRV	D1546	France	2010	Muscovy duck	KJ871017–KJ871026
	ZJ2000M	China, Zhejiang	2011	Muscovy duck	KF306082–KF306091
	D2044	France	2012	Muscovy duck	KJ871007–KJ871016
NMDRV	J18	China, Fujian	2008	Muscovy duck	JX478260–JX478269
	NP03	China	2009	Muscovy duck	KC312699–KC312702 JF320800–JF320803 GU338025, GQ888710
	ZJ00M	China, Zhejiang	2000	Muscovy duck	KF154110–KF154119
	SH12	China, Guangdong	2012	Muscovy duck	MH510245–MH510254
	SD‐12	China, Shandong	2012	Mallard wild duck	KJ879924–KJ879933
	DH13	China	2013	Muscovy duck	MH510255–MH510264
GRV	D20‐99	Hungary	1999	Goose	KF809662–KF809671
NGRV	03G	China	2003	Goose	JX145328–JX145337

*Note*: Strains marked with  ^∗^ in the upper right corner are isolates from this study. XT18 and LY20 were sequenced in our previous studies, and the other eight isolates were newly sequenced here.

### 2.2. Viral Whole‐Genome Sequencing and Assembly

Viral RNA was extracted using the Total Viral RNA Isolation Kit. Sequencing libraries were constructed with the TruSeq RNA Sample Preparation Kit. Following mRNA purification, RNA was fragmented and reverse‐transcribed to synthesize first‐strand cDNA, followed by second‐strand cDNA synthesis. After end repair, A‐tailing, and Illumina adapter ligation, the libraries were enriched by PCR amplification. Purified libraries were subjected to paired‐end sequencing on the HiSeq platform by Shanghai Personal Biotechnology Co., Ltd. Raw reads were processed to remove adapters and nontarget sequences such as ribosomal RNA and then de novo assembly using CLC Genomic Workbench V7.5. The resulting contigs were aligned against reference genomes and refined by remapping raw reads, and consensus sequences were finally extracted.

### 2.3. Sequence Analysis

The obtained NDRV sequences were edited, assembled, and corrected using EditSeq from the DNASTAR Lasergene 12 Core Suite (DNASTAR, Inc.), and predicted open reading frames (ORFs) with corresponding deduced aa sequences. Sequence homology assessments were performed using BLASTN online (http://blast.ncbi.nlm.nih.gov/Blast.cgi) against published sequences. nt and aa identity alignments were conducted using Clustal W in MegAlign. aa disorder analysis was done using Jalview desktop Version 2.11.5.2.

### 2.4. Phylogenetic Analysis

We constructed phylogenetic trees using MEGA V7.0 for all 10 gene‐segment sequences from this study and the corresponding reference sequences (Table 1). Trees were generated with the neighbor‐joining method, and branch node confidence was assessed via 1000 bootstrap replicates.

### 2.5. Whole‐Genome Visualization Analysis

Whole‐genome visualization analysis was performed using the mVISTA online platform (http://genome.lbl.gov/vista/mvista/submit.shtml). Sequence identity curves were generated to display conserved regions and variable hotspots among different strains, with pink regions indicating identity ≥95% and white regions indicating identity <95%.

### 2.6. Recombination Analysis

Recombination signal scanning across the NDRV whole‐genome sequences was performed using the recombination detection program (RDP5, Version 5.5) and similarity plotting (Simplot). In RDP5, we simultaneously applied seven detection algorithms: RDP, GENECONV, MaxChi, Chimaera, SiScan, LARD, and 3Seq. To control false positives, we considered a gene segment as harboring a credible recombination event only when detected by five or more algorithms with a *p*‐value < 10^−14^. Subsequently, maximum likelihood (ML) phylogenetic trees were constructed in MEGA V7.0 to confirm precise breakpoints and likely parental sources.

### 2.7. Pathogenicity Analysis Against Spleen

We selected three representative NDRV isolates (XT18, SDLS, and SDYC) and administered them at the same dose (10^5^ TCID_50_ per duckling) via intravenous injection to 1‐day‐old Cherry Valley ducklings. The ducklings were purchased from a commercial hatchery in Tai’an, Shandong Province. Before inoculation, serum and cloacal swab samples were collected and tested by qPCR to confirm that the ducklings were free of NDRV and other duck pathogens, and serum samples were confirmed negative for NDRV antibodies using an indirect ELISA. At 1, 2, 3, 5, 7, 9, 11, 13, 15, 17, 19, and 21 days postinfection (dpi), three experimental ducklings were randomly selected from each group. Prior to euthanasia, ducklings were rendered deeply unconscious via inhalation of high‐concentration CO_2_, followed immediately by intravenous exsanguination to ensure rapid, humane death. Following euthanasia, spleen tissues were collected, fixed in 10% neutral formalin, paraffin‐embedded, sectioned, and stained with hematoxylin–eosin (HE) for histopathological examination. We extracted total RNA from spleens and quantified viral loads using a TaqMan probe‐based real‐time quantitative PCR assay previously established [24], with primer and probe as follows: NDRV‐F: CCCGGATTCTCGATGAATGGT, NDRV‐R: CGACCCACTGCTGGATACAAG, NDRV‐probe: FAM‐AACGCCTGTGCACGAGCTGAAC‐TAMRA. Fluorescence signals were analyzed using LightCycler96 SW 1.1 software. At each time point, three ducklings per group were sampled as three biological replicates, and the extracted total RNA was diluted to the same concentration prior to quantification. Viral loads are expressed as viral genome copies per μg of total RNA (copy numbers/μg RNA), and data are presented as the mean ± standard deviation (SD). Statistical analyses were performed using GraphPad Prism 9.0 (GraphPad Software, USA).

## 3. Results

### 3.1. Genomic Characteristics of the Isolates

Ten NDRV strains isolated between 2018 and 2023 were analyzed in this study. Eight of them were newly sequenced, while the genomes of XT18 and LY20 were previously deposited by our team and were reanalyzed here. The assembled full‐length genome was 23,418 bp, comprising 10 linear dsRNA segments, ranging from 1191 bp (S4) to 3959 bp (L1). All segments had the ARV species‐specific conserved terminal sequences at both ends (5^′^‐GCUUUU and 3^′^‐UCAUC). Except for the tricistronic S1 gene, the remaining nine segments were monocistronic, encoding a total of 12 viral proteins from 98 aa (p10) to 1294 aa (λA). Coding assignments matched those of other known NDRV strains.

### 3.2. Sequence Identity Analysis

To assess the genetic diversity, we performed nt and deduced aa sequence homology analyses on the complete genome sequences. The nt identity among our isolates ranged from 86.9% to 99.9% and aa identity from 94.8% to 100.0% across all segments (Table 2). However, variation varied markedly among segments. Specifically, the L2 (nt 93.3%–95.3%), M1 (nt 94.3%–97.1%), S2 (nt 86.9%–96.4%), S3 (nt 95.3%–98.2%), and S4 (nt 93.9%–97.2%) showed relatively lower nt homologies, with their encoded proteins (λB, μA, σA, σB, and σNS) also showing corresponding aa variations. Notably, the σC encoding gene, which is traditionally considered a hypervariable region, showed nt and aa identities of 97.2%–99.6% and 97.2%–99.7%, respectively, in this study, not exhibiting the expected higher variability. These results indicate that, in addition to the traditionally hypervariable S1 gene, other segments such as L2, M1, S2, and S4 also harbor considerable sequence diversity among currently circulating NDRV strains. Whether this variability reflects differential selection pressure requires formal selection analysis in future studies.

**Table 2:** Sequence identities between NDRV isolate SDYC and other waterfowl‐derived orthoreovirus strains.

Strain	Highest identity NDRV strain			Among all NDRV strains		Among DRV strains		Highest identity other strain
	Strain	nt	aa	nt	aa	nt	aa	(nt/aa)
λA	XT18	99.2	99.8	96.6–99.2	99.4–99.8	97.1–98.0	99.1–99.8	SH12 (98.6/99.8)
λB	XT18	95.3	97.9	92.9–95.3	97.3–97.9	93.6–93.8	97.9	NP03 (94.1/97.6)
λC	SDWF21	99.0	99.5	96.8–99.0	98.8–99.7	96.5–98.4	98.8–99.2	ZJ00M (98.6/99.3)
μA	XT18	97.1	99.0	94.3–97.1	96.3–99.0	95.5–96.5	98.4–98.5	ZJ00M (95.7/98.2)
μB	XT18	99.7	99.7	96.8–99.7	97.9–100	96.8–98.2	97.8–98.8	SD‐12 (98.0/98.5)
μNS	SDWF21	99.9	100	96.0–99.9	97.6–100	97.8–98.3	98.4–98.9	ZJ00M (98.4/98.7)
p10	XT18/SDLC23/SY	99.9	100	95.9–99.0	96.9–100	96.6–97.6	98.0–99.0	SD‐12 (98.0/99.0)
p17	JSSQ22/SDLH23	99.0	99.4	96.3–99.0	95.1–99.4	96.3–97.8	95.7–96.3	NP03(97.8/95.7)
σC	XT18/JSSQ22/SY	99.6	99.7	95.0–99.6	94.1–99.7	95.7–97.9	96.7–98.8	SD‐12 (98.2/97.8)
σA	XT18	96.4	98.8	86.7–96.4	96.6–98.8	86.1–95.6	97.1–98.6	SH12 (95.9/98.6)
σB	SDWF21	98.2	98.6	95.3–98.2	94.8–98.6	96.1–97.5	96.5–98.4	SH12 (97.3/98.4)
σNS	SDWF21	97.3	99.7	93.9–97.1	98.9–99.7	91.8–96.5	97.0–99.5	SH12 (96.0/99.5)

*Note*: “nt” represents nucleotide sequences, while “aa” denotes amino acid sequences. Screening for the strain with the highest identity is primarily based on nucleotide sequence identity. The term “highest identity with other strains” specifically refers to the identity comparison between the SDYC strain and isolates of MDRV, NDRV, GRV, and NGRV.

Comparison of our isolates with domestic and international reference strains revealed nt identities of 86.7%–99.9% and aa identities of 94.1%–100.0% across all gene segments. Against representative DRV strains, the nt and aa identities were 86.1%–98.4% and 95.7%–99.8%, respectively, further substantiating the marked genetic divergence between NDRV and classical DRV. Taking isolate SDYC as an example, its different gene segments showed the highest sequence identity with strains of different origins, such as XT18 and SDWF21. This mosaic distribution provides fresh evidence for frequent reassortment during NDRV evolution.

### 3.3. Phylogenetic Analysis

To investigate the phylogenetic relationships, we built trees based on the nt sequences of all 10 segments (Figure 1). In general, for L and M genes, NDRV isolates, both ours and reference strains, clustered together, with clear genetic differentiation from classical DRV, MDRV, and GRV. However, some NDRV isolates clustered closely with classical strains in specific segments. For instance, strain SD19‐6201 clustered tightly with the DRV isolate TH11 at the L1 gene. Strain JS0122 clustered closely with the MDRV isolate J18 across multiple gene segments (L1, L2, M1, and M2) but was genetically distant from other NDRV isolates in the remaining gene segments. This topological incongruence among segment‐specific phylogenies suggests that JS0122 may have arisen through reassortment between the NDRV and MDRV lineages. A similar pattern was observed for SD19‐6201. At the S1 locus, all NDRV isolates except JS0122 and SD19‐6201 formed a highly clustered group. In contrast, certain NDRV isolates formed independent phylogenetic lineages at the S2, S3, and S4 gene, namely, SDYC (S2), JS0122 (S3), and SDLH23 (S4), which may also reflect reassortment or strong lineage‐specific selection in these segments.

**Figure 1:**
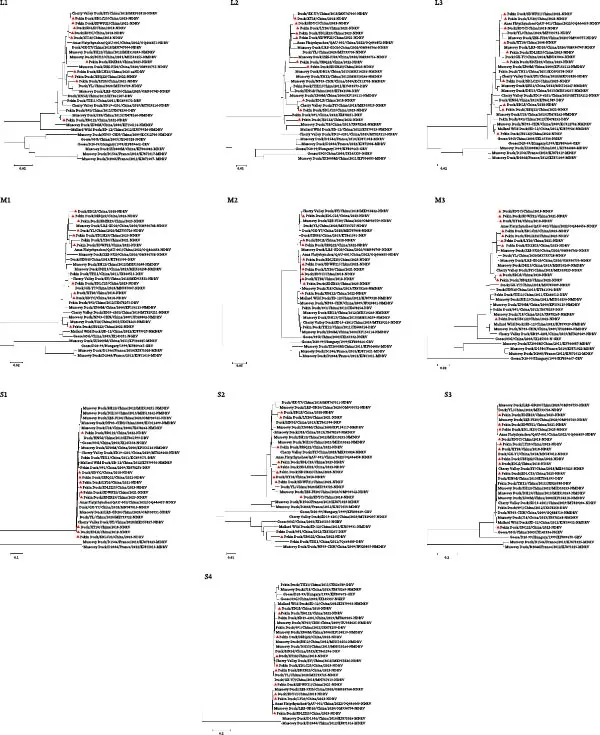
Phylogenetic analysis of waterfowl‐derived orthoreovirus. The NDRV isolates obtained in this study are marked with red triangles.

### 3.4. Whole‐Genome Visualization Analysis

We performed whole‐genome identity comparisons between our isolates and reference strains using mVISTA. The overall similarity was high across the entire genome, indicating that the NDRV genome is highly conserved (Figure 2). Nevertheless, we observed significant variations (identity < 95%) in the L2, M1, S2, and S4 segments among different strains, pointing to local mutation hotspots. Several strains, including GX‐Y7, SD19‐6201, and SDWF21, showed some degree of sequence divergence from the main group in multiple segments. Overall, the NDRV genomic backbone is highly conserved, but specific gene segments display considerable sequence diversity, which may relate to immune evasion and adaptive evolution.

**Figure 2:**
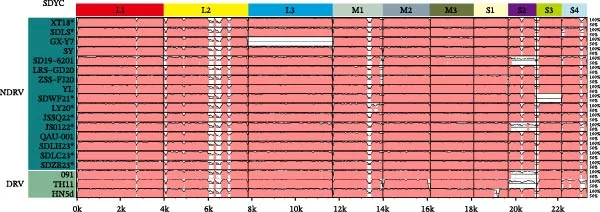
Whole‐genome visualization analysis using mVISTA. Pink areas represent ≥95% identity, white areas represent <95% identity. The scale bar indicates the approximate length of the concatenated genome.

### 3.5. aa Variation Analysis

In the four proteins λB, μA, σA, and σNS, known functional motifs, including the RNA polymerase motif in λB and the dsRNA‐binding active sites in σA, remained intact, with no aa substitutions or deletions detected. In contrast, the σC protein exhibited marked aa sequence diversity. Within its antigen‐binding and cell receptor‐binding regions, the newly isolated strains SDYC, SDWF21, LY20, JS0122, SDLH23, and SDZB23 all had aa mutations to varying degrees. Notably, JS0122 carried 6 and 4 aa variations in these two functional regions, respectively, while the reference strain ZSS‐FJ20 showed 9 and 10 variations. Of particular interest, JS0122 and ZSS‐FJ20 shared a highly consistent pattern of aa substitutions at multiple positions (Table 3).

**Table 3:** Amino acid variations in NDRV isolates at the conserved functional motifs of σC, λA, μA, σA, and σNS proteins.

Protein	Conserved motifs with different biological functions	Amino acid variant sites		
		Strain	Number	Difference site of aa
λB	RNA polymerase (^522^RxxRxI^527^, ^584^DxxxxDxS^591^, ^680^PSGxxxT^686^, ^732^GDD^734^)	—	—	—
μA	ATPase conserved ^(403^GLCMPKGSFKS^413^) N6‐adenine‐specific DNA methyltransferase (^458^LALDPPF^464^)	—	—	—
σC	Antigenicity‐related (22–76, 119−177) Receptor binding (151–326)	SDYC ^∗^	1	298: V‐A
		SDWF21 ^∗^	2	36: D‐E, 151: T‐M
		LY20 ^∗^	2	151: T‐M, 212: I‐L
		JS0122 ^∗^	8	132: A‐S, 138: R‐Q, 149: A‐V, 150: P‐L, 152: Q‐R, 158: H‐Q, 253: A‐V, 277: F‐R
		SDLH23 ^∗^	2	138: R‐Q, 306: T‐I
		SDZB23 ^∗^	3	199: S‐A, 212: I‐L, 293: S‐A
		GX‐Y7	1	199: S‐A
		SD19‐6201	5	63: T‐I, 74: S‐G, 138: R‐Q, 158: H‐Q, 253: A‐V, 306: T‐I, 310: Q‐R
		LRS‐GD20	1	199: S‐A
		ZSS‐FJ20	14	55: T‐M, 57: A‐T, 132: A‐S, 138: R‐Q, 149: A‐V, 150: P‐L, 152: Q‐R, 158: S‐Q, 161: N‐D, 183: A‐T, 223: M‐L, 226: Q‐P, 227: N‐T, 247: I‐M, 253: A‐V, 310: Q‐L
		YL	1	151: T‐M, 200: G‐D, 310: Q‐R
		QAU001	2	25: T‐M, 132: A‐S, 151: T‐M, 200: G‐D, 310: F‐R
σA	dsRNA‐binding active site motif (^155^R–R^172^)	—	—	—
σNS	ssRNA binding arginine residues (R^6^, R^11^, R^234^, R^287^, R^365^)	—	—	—

*Note*: Strains marked with  ^∗^ in the upper right corner are isolates from this study.

Further analysis of the spatial distribution of variant sites revealed that the region spanning aa 85–158 of the σC constitutes a hypervariable domain (Figure 3). In λB, variations are primarily in the N‐terminal residues 11–50. Strain LY20 harbors a high density of variant sites in μA. Variations in σA are distributed randomly across the full‐length sequence, whereas variations in σNS are relatively clustered in the C‐terminal region spanning residues 321–340 (Supporting Information: Figures S1–S4).

**Figure 3:**
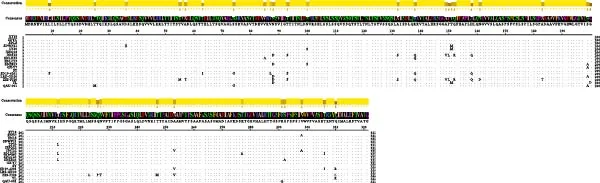
Amino acid sequence variation in the σC protein of NDRV isolates.

### 3.6. Recombination Analysis

As a segmented RNA virus, NDRV can undergo both intrasegment recombination and segment reassortment during mixed infections. Our recombination analysis revealed that strains XT18 and LY20 each carried intrasegment recombination events. Specifically, the M2 segment of XT18 was identified as a recombinant sequence, with SDYC as the major parent and HN5d as the minor parent (Figure 4A). The S4 segment of LY20 was likewise a recombinant, with GX‐Y7 as the major parent and SDYC as the minor parent (Figure 4B). All these recombination events met the statistical criteria of positive detection by at least five recombination algorithms with *p*‐values < 10^−14^ (Table 4), whereas no reliable recombination signals were detected in the other segments.

**Figure 4:**
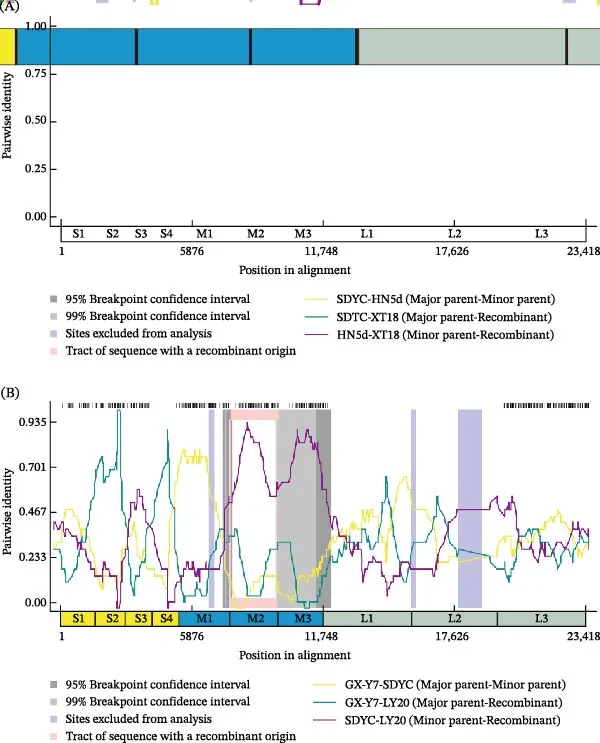
Reassortment Analysis of NDRV Isolates XT18 and LY20. (A) Reassortment analysis between strain XT18 and other isolates (B) Reassortment analysis between strain LY20 and other isolates.

**Table 4:** Possible recombination events present in NDRV XT18 and LY20 strains.

Strain	Gene segment	Major parent (similarity %)	Minor parent (similarity %)	Detection methods (*p*‐value)
XT18	M2	SDYC (99%)	HN5d (99.2%)	RDP (1.424 × 10^−15^) GENECONV (3.023 × 10^−14^) MaxChi (4.379 × 10^−16^) Chimaera (3.679 × 10^−16^) 3Seq (3.093 × 10^−17^)
LY20	S4	GX‐Y7 (98.4%)	SDYC (99.3%)	RDP (8.538 × 10^−15^) GENECONV (1.858 × 10^−15^) MaxChi (1.967 × 10^−14^) Chimaera (5.737 × 10^−15^) SiScan (4.404 × 10^−15^) 3Seq (5.307 × 10^−10^)

### 3.7. Pathogenicity Analysis Against Spleen

Pathogenicity analysis in duckling spleens revealed that XT18 infection caused relatively mild splenic lesions, with only scattered necrotic foci. In contrast, SDLS and SDYC induced severe splenic necrosis at 7 dpi and 5 dpi, respectively, with necrotic lesions almost covering the entire splenic surface. By 15 dpi, necrotic symptoms had markedly improved in all groups (Figure 5A). Histopathological examination revealed significant differences in the extent and progression of splenic damage among the three isolates. XT18 showed the weakest pathogenicity, with no obvious lesions at 2 dpi, necrosis of white pulp lymphoid follicles with architecture that remained discernible at 5 dpi, splenic sinus dilation and lymphocyte depletion at 7 dpi, and only mild congestion and macrophage infiltration with gradual recovery by 15 dpi. SDLS presented with splenic pulp congestion, fibrinoid exudation, and lymphocyte necrosis at 2 dpi, aggravated congestion and structural obliteration at 5 dpi, tissue necrolysis with neutrophilic infiltration at 9–11 dpi, and persistent congestion and macrophage infiltration at 15 dpi. SDYC exhibited the most severe splenic pathology, with severe splenic pulp congestion and extensive fibrinoid exudation at 2 dpi, tissue necrolysis at 5 dpi, marked lymphocyte depletion and vascular wall thickening at 7 dpi, granuloma formation at 9 dpi, and replacement of necrotic areas by fibrous tissue at 15 dpi, representing the longest duration of lesions (Figure 5B). Dynamic viral load monitoring showed that in the early phase, all three strains were detectable with an upward trend. During the middle and late phases, SDYC maintained relatively higher viral loads, being significantly higher than XT18 at 7 dpi (*p* = 0.028) and significantly higher than both XT18 and SDLS at 13 dpi (*p* = 0.0003 and *p* = 0.0006, respectively) (Figure 5C). This indicates that SDYC may have a greater adaptive capacity for sustained replication in the spleen.

**Figure 5:**
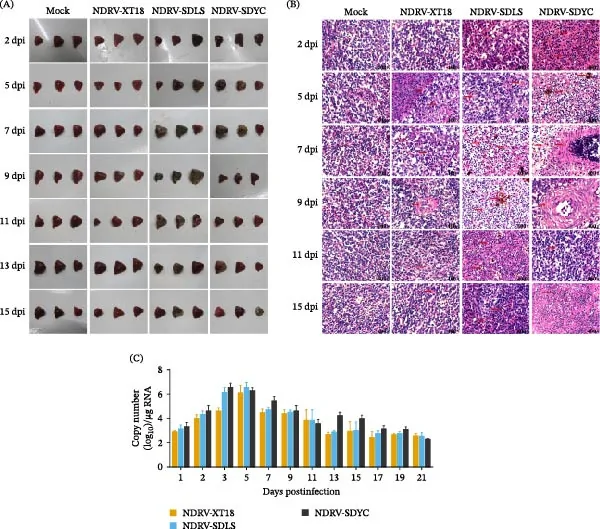
Splenic pathological damage induced by NDRV infection in ducklings. (A) Gross pathological changes in the spleens of ducklings following NDRV infection. (B) Histopathological changes in the spleens of ducklings following NDRV infection. No significant pathological changes were observed in the control group from 2 to 15 dpi. XT18‐infected group: at 2 dpi, no obvious pathological changes were evident; at 5 dpi, lymphoid follicle necrosis was observed in the splenic white pulp, with relatively clear demarcation between the white and red pulp; at 7 dpi, splenic sinus dilation was noted, with reduced lymphocyte necrosis and loss of clear splenic architecture; at 9 dpi, lymphocyte necrosis persisted with red pulp cell necrosis and vascular wall thickening; at 11 dpi, splenic sinus dilation and reticular cell hyperplasia were observed; and at 15 dpi, mild splenic congestion and macrophage infiltration were present. SDLS‐infected group: at 2 dpi, splenic pulp congestion, fibrinoid exudation, and lymphocyte necrosis were evident; at 5 dpi, splenic congestion, lymphocyte necrosis and disintegration, reduced red pulp cell numbers, loss of structural integrity, and macrophage infiltration were observed; at 7 dpi, diminished lymphocyte necrosis was accompanied by splenic corpuscle and cord atrophy, with substantial fibrinoid exudation and inflammatory cell infiltration; at 9 dpi, tissue necrosis and disintegration, congestion, and neutrophilic infiltration were seen; at 11 dpi, congestion and neutrophilic infiltration persisted; and at 15 dpi, splenic congestion and macrophage infiltration were noted. SDYC‐infected group: at 2 dpi, severe splenic pulp congestion was accompanied by abundant homogeneously eosinophilic fibrinoid exudate, with scattered neutrophils and lymphocytes; at 5 dpi, focal tissue necrosis and disintegration, congestion, lymphocytolysis, and neutrophilic infiltration were observed; at 7 dpi, extensive tissue necrosis and disintegration with marked lymphocyte depletion due to necrosis, vascular wall thickening, and abundant lymphocytes aggregated along the inner vascular wall were seen; at 9 dpi, focal tissue necrosis with granuloma formation was evident; at 11 dpi, splenic sinus dilation, focal necrosis and disintegration, and macrophage infiltration were present; and at 15 dpi, necrotic areas of the spleen were replaced by fibrous tissue, with scattered lymphocytes. (C) Dynamic changes in splenic viral loads in ducklings postinfection, as determined by quantitative real‐time PCR. Three ducklings were sampled at each time point as biological replicates. Total RNA was normalized to the same concentration, and data are presented as mean ± SD (copy numbers/μg RNA).

## 4. Discussion

The ongoing prevalence and evolution of NDRV pose a persistent threat to China’s waterfowl industry, making the dynamic surveillance of genetic variation, reassortment, and pathogenicity changes critical. Here, we conducted whole‐genome phylogenetic analysis, reassortment detection, and pathogenicity evaluation in ducklings for Chinese NDRV isolates. We identified L2, M1, S2, and S4 as variable hotspot genes, confirmed statistically significant recombination events in the M2 gene of XT18 and the S4 gene of LY20, and found marked differences in splenic pathogenicity and viral replication capacity among isolates (SDYC, SDLS, and XT18). These findings not only enrich the molecular epidemiological data for NDRV but also lay a foundation for understanding the mechanistic links between viral evolution and pathogenicity.

The continuous evolution of key gene segments represents a crucial strategy for the virus to breach host immune barriers and sustain circulation [25, 26]. Different NDRV gene segments may have been subjected to divergent selection pressures, with L2, M1, S2, and S4 emerging as new diversity‐rich regions. The traditional view holds that the S1 gene is the most variable segment, as its encoded σC protein is directly exposed to host immune selection [27]. In our isolates, S1 showed nt homologies of 97.2%–99.6%, and aa variation analysis revealed extensive mutations in σC within both the antigen‐binding and receptor‐binding domains, with the region spanning residues 85–158 showing particularly high variability. Multiple isolates shared a highly consistent pattern of aa substitutions, suggesting that specific mutation combinations may be under positive selection during adaptive evolution. Furthermore, the L2, M1, S2, and S4 segments showed more pronounced diversity. This suggests that beyond the traditional immune‐related genes, segments encoding proteins involved in replication, transcription, or assembly may also accumulate considerable sequence diversity during sustained NDRV prevalence, although the underlying evolutionary forces remain to be determined by formal selection analysis. Existing studies estimate that the average evolutionary rate of ARV is ~2.3 × 10^−3^ substitutions per site per year [28], this relatively rapid rate may indeed underline the differential variation among gene segments. The evolutionary rate differences and their functional implications warrant quantitative assessment in future studies.

Both intrasegment recombination and segment reassortment contribute to the evolution of orthoreoviruses. In this study, topological incongruence among segment‐specific phylogenies suggested possible reassortment in isolates such as JS0122 and SD19‐6201, whereas RDP5 analysis identified recombination events in the M2 segment of XT18 and the S4 segment of LY20. All recombination events met the criteria of detection by at least five algorithms with *p*‐values < 10^−14^, ensuring high statistical reliability [29]. Furthermore, our homology analysis yielded a key insight, taking SDYC as an example, different gene segments share the highest sequence identity with distinct donor strains, such as XT18 and SDWF21, and this mosaic distribution itself constitutes indirect evidence for recombination [19]. Specifically, the M2 segment of XT18 had SDYC as the major parent and HN5d as the minor parent, while the S4 segment of LY20 had GX‐Y7 as the major parent and SDYC as the minor parent. Compared with previously reported recombination or reassortment events in waterfowl orthoreoviruses, which involved the μB‐ and σC‐encoding genes of classical DRV isolate TH11 [18], multiple host‐derived viruses in the German isolate D2533/4/1‐10 [19], and the L1 segment of NDRV isolate FJ19 [20], the M2 and S4 segments identified here have not been previously associated with recombination in NDRV. In addition, the parental strains involved in our events were all of Chinese origin and are currently cocirculating in domestic ducks, suggesting that recombination is occurring among local NDRV strains rather than involving geographically distant or cross‐species donors. Notably, SDYC was confirmed in this study as the most pathogenic strain and also served as a parental strain in both recombination events, and HN5d has been previously reported to carry an 18‐aa deletion in its σC linked to high pathogenicity [14]. Whether the integration of genetic material from these highly pathogenic strains directly contributes to the pathogenic characteristics of XT18 and LY20 warrants further investigation.

The host range of NDRV continues to expand, with recent reports increasingly breaking down traditional host barriers [20, 22, 30], and genetic reassortment may be a key factor driving this expansion [31, 32]. The newly isolated reassortment strain HZ01/22 has been confirmed to infect chickens and replicate effectively, with partial lethality [4], contrasting sharply with the conventional view that NDRV has low pathogenicity in chickens. More importantly, natural reassortment events between waterfowl‐ and chicken‐origin orthoreovirus have been clearly documented, confirming active gene exchange at the waterfowl–poultry interface [33, 34]. Accumulating evidence also supports the role of Anseriformes wild birds as potential reservoir hosts. DRV nucleic acid has been detected in feces from wild ducks, egrets, and swans in eastern China’s coastal wetlands [22], and Australian researchers have further reported NDRV infection in Pacific black ducks, suggesting transregional spread via the East Asia‐Australasia migratory pathway [30]. The 10 NDRV isolates in this study were primarily collected from Shandong and Jiangsu, which are key nodes along migratory bird routes. Therefore, the risk of virus introduction through wild waterfowl and subsequent sustained transmission and reassortment in domestic poultry cannot be overlooked, and continued surveillance at the wild bird–domestic poultry interface is warranted.

Pathogenicity varies considerably among NDRV isolates and correlates closely with genetic characteristics and replication capacity [3, 35]. In our duckling infection experiments, SDYC caused the most severe splenic lesions and maintained the highest viral loads throughout the middle and late stages of infection among the three tested strains, indicating a stronger ability to induce splenic damage and sustain replication in this target organ. It should be noted, however, that virulence here was inferred from splenic pathology and viral load dynamics rather than being quantitatively compared through comprehensive lesion scoring. Previous studies have indicated that although reassortment between NDRV and classical DRV is common, significant genetic linkage may exist among the gene segments encoding μB, σB, and σC proteins, with specific gene combinations codistribution with pathotypes [21]. In this study, we found that aa variations in SDYC are primarily concentrated in the N‐terminal region of λB (residues 11–40), which has accumulated extensive substitutions. Given that λB serves as the core RNA‐dependent RNA polymerase mediating transcription and replication, mutations in this region could modulate virulence by altering the enzyme activity or protein–protein interactions. However, this hypothesis requires validation via reverse genetics. NDRV infection can induce immunosuppression in ducks, increasing the risk of secondary bacterial infections such as *Escherichia coli* and *Riemerella anatipestifer* [27, 36]. However, coinfection does not always lead to aggravated lesions [37]. The varying degrees of splenic damage induced by different strains may directly modulate the host’s immune response and consequently affect the occurrence and severity of secondary infections.

This work reports, for the first time, the recombination events and parental origins of the M2 and S4 segments in NDRV, and reveals significant pathogenicity differences among different NDRV isolates. These findings provide important fundamental data for molecular epidemiological surveillance. Future efforts should focus on continued surveillance of NDRV transmission chains, with particular attention to host range dynamics and cross‐species transmission events. The molecular mechanisms underlying viral evolution, especially how specific gene combinations determine virulence and pathotype, merit in‐depth investigation. Strengthening biosecurity measures on farms and reducing mixed infections are essential to minimize viral reassortment events at the source. Only through systematic integration of molecular surveillance, functional validation, and field‐based control measures can effective management of NDRV be achieved in the context of continuous viral evolution.

## Author Contributions

**Chenchen Jiang**: investigation, visualization, writing – original draft. **Rongchang Liu**: investigation, visualization, writing – original draft. **Bing Li**: investigation, visualization. **Wenzhe Liu**: investigation, data curation. **Di Lei**: visualization, software. **Yu Huang and Chunhe Wan**: methodology, resources, data curation. **Yi Tang**: conceptualization, data curation, funding acquisition. **Rendong Fang**: funding acquisition, writing – review and editing. **Hongzhi Wang**: conceptualization, funding acquisition, writing – review and editing.

## Funding

This study was supported by grants from the Yunnan Province Major Science and Technology Special Project (Grant 202602AE090011), the Chongqing Technology Innovation and Application Development Project (Grant CSTB2025TIAD‐GPX0004), the Program of Fujian Provincial Key Laboratory for Avian Diseases Control and Prevention (Grant FKADL‐2025‐02), the Chongqing Modern Agricultural Industry Technology System (Grant CQMAITS202614), and the earmarked fund for the China Agriculture Research System (Grant CARS‐42‐19).

## Disclosure

All authors have read and approved the final version of the manuscript.

## Ethics Statement

All animal experiments were performed strictly in accordance with the ARRIVE guidelines and approved by the Animal Ethics Committee of Southwest University (Approval Number LAC2024‐1‐0266). The animal experimental subjects were 1‐day‐old domestic ducklings. Written informed consent was obtained from the animal owners after full explanation of the purpose and procedures of this research prior to the study.

## Conflicts of Interest

The authors declare no conflicts of interest.

## Supporting Information

Additional supporting information can be found online in the Supporting Information section.

## Supporting information


**Supporting Information 1** Figure S1. Amino acid sequence variation in the λB protein of NDRV isolates Supplementary.

**Supporting Information 2** Figure S2. Amino acid sequence variation in the μA protein of NDRV isolates.

**Supporting Information 3** Figure S3. Amino acid sequence variation in the σA protein of NDRV isolates.

**Supporting Information 4** Figure S4. Amino acid sequence variation in the σNS protein of NDRV isolates

## Data Availability

All data and results from this study are publicly available. Raw data and Supporting Information supporting the findings have been deposited in Figshare repository (https://doi.org/10.6084/m9.figshare.33363789). The complete genome sequences of the NDRV isolates generated in this study have been deposited in the NCBI (https://www.ncbi.nlm.nih.gov/nuccore/) under the Accession Number MK749398 and other associated accession numbers, all of which are listed explicitly in Table 1.
